# Supplementary material for: A Comparison of Two Versions of the CRISPR-Sirius System for the Live-Cell Visualization of the Borders of Topologically Associating Domains
Source: Cells. 2024 Aug 27;13(17):1440. doi: 10.3390/cells13171440 (PMC11394217; doi:10.3390/cells13171440)
Supplement: Supplementary file 1 [file cells-13-01440-s001.zip › Supplementary Figures.pdf]

cells

MDPI

Supplementary Information

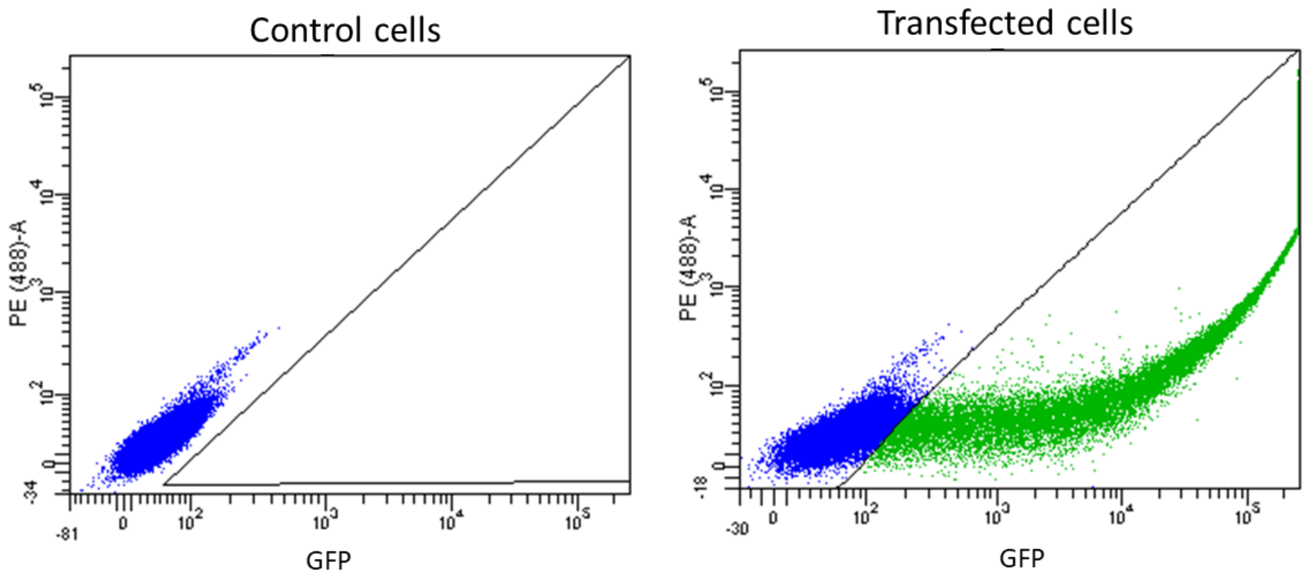

Figure S1. Flow cytometry analysis of transiently transfected cells. Representative dot plots for untransfected cells (left) and cells transfected with a plasmid encoding MCP-sfGFP (right). The analysis was performed 48 hours after transfection. The proportion of GFP^+cells (triangular area on the right of each dot plot) after transfection averaged 40.2% for three technical replicates (replicate results: 41.9%, 38.2%, and 40.4%). PE fluorescence was measured upon excitation with a 488 nm laser to distinguish true GFP^+cells from highly autofluorescent cells (those with moderately high values of both PE and GFP fluorescence; such cells fall close to the diagonal on the dot plot).

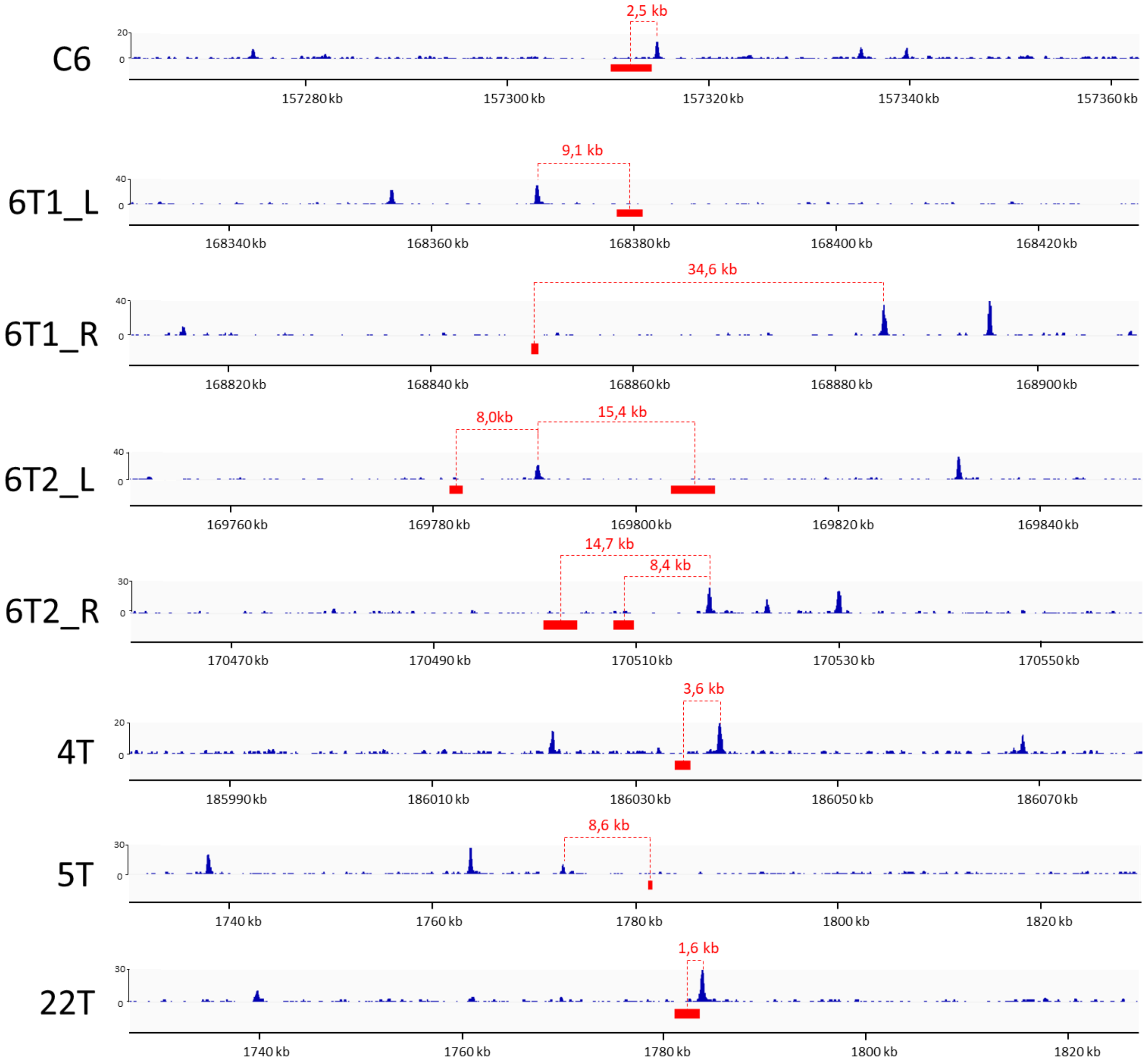

Figure S2. ChIP-Seq profiles (fold enrichment) for the RAD21 cohesin subunit around the borders of TADs selected in this study. Red rectangles denote the positions of clusters of visualizing sgRNA binding sites. The distances from the midpoints of sgRNAs clusters to the nearest RAD21 peaks are shown in red. The coordinates are for the hg18 human genome assembly.

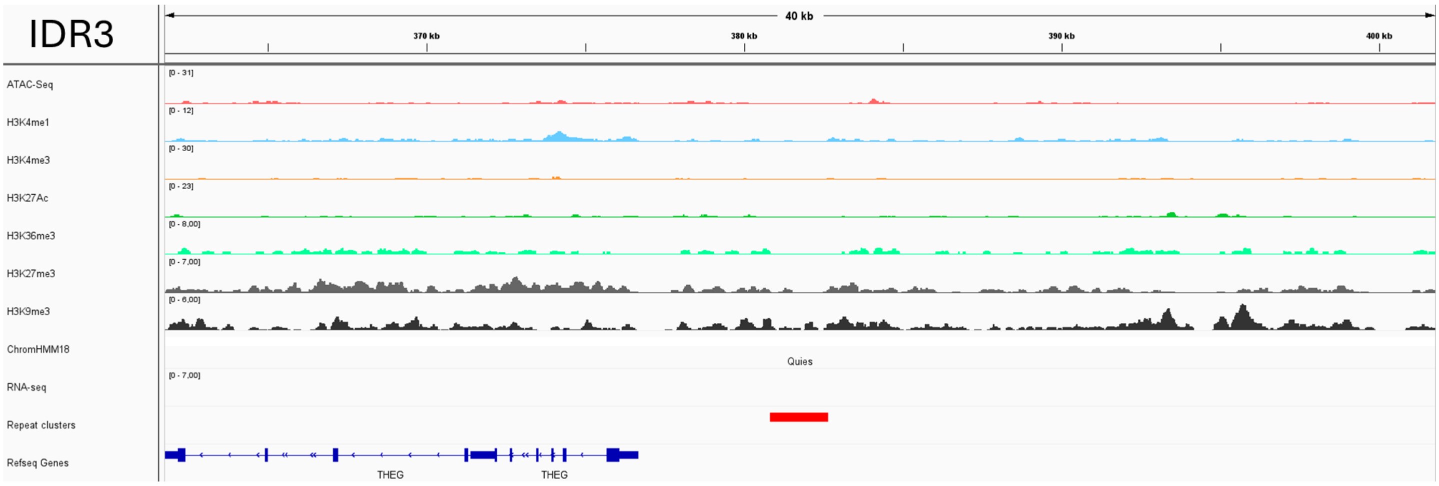

Figure S3. Epigenetic context of the IDR3 locus (Chr19). Shown are tracks for ATAC-Seq (fold change over control), the specified histone marks (fold change over control), ChromHMM chromatin 18-state model and PolyA-plus RNA-seq (signal of unique reads). See "Material and Methods" for the references to the datasets). Minimal and maximal values for each track are shown on the left in linear scale (the same scale for Figures S3-S11). Red rectangle corresponds to the position of the IDR3 repeat cluster. Genomic coordinates are for hg38 human genome assembly.

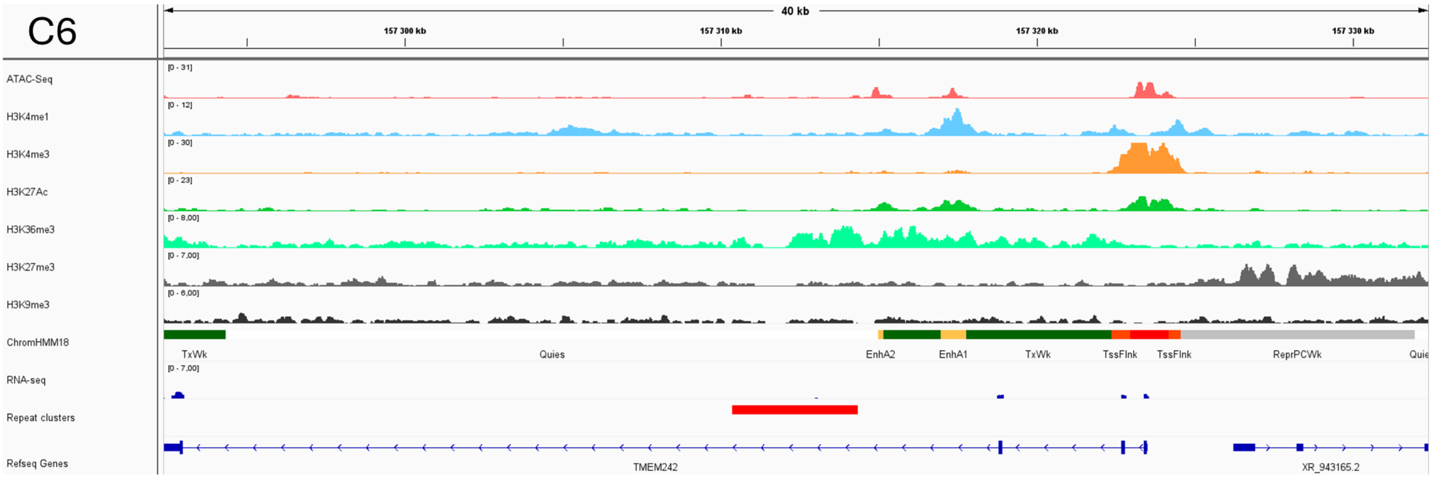

Figure S4. Epigenetic context of the C6 locus (Chr6). Shown are tracks for ATAC-Seq (fold change over control), the specified histone marks (fold change over control), ChromHMM chromatin 18-state model and PolyA-plus RNA-seq (signal of unique reads). See "Material and Methods" for the references to the datasets). Minimal and maximal values for each track are shown on the left in linear scale (the same scale for Figures S3-S11). Red rectangle corresponds to the position of the C6 repeat cluster. Genomic coordinates are for hg38 human genome assembly.

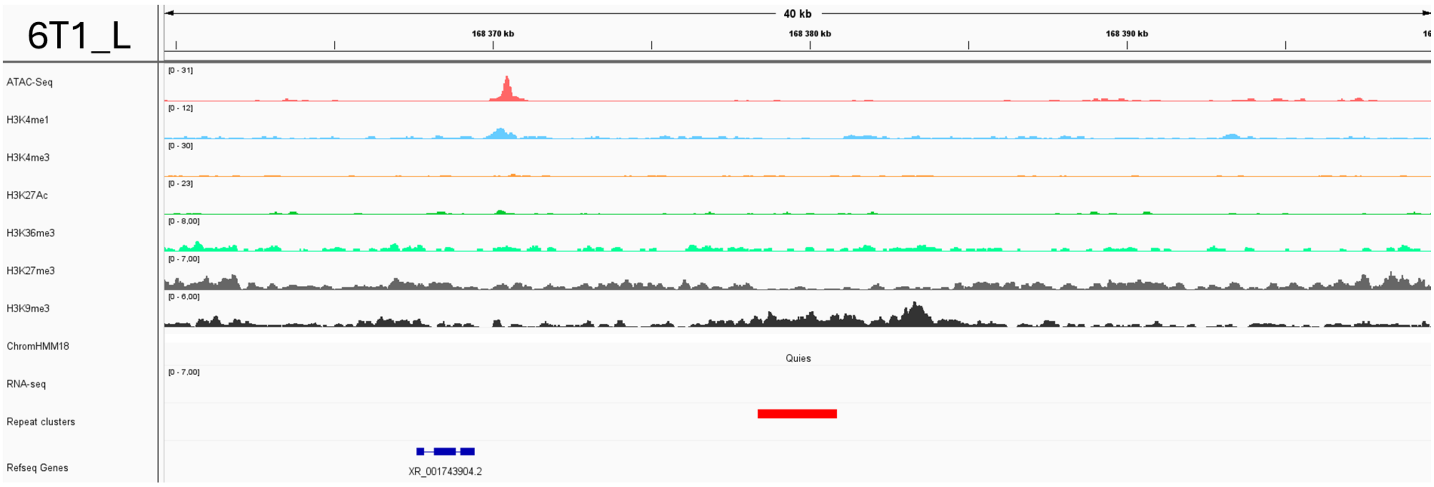

Figure S5. Epigenetic context of the 6T1_L locus (Chr6). Shown are tracks for ATAC-Seq (fold change over control), the specified histone marks (fold change over control), ChromHMM chromatin 18-state model and PolyA-plus RNA-seq (signal of unique reads). See "Material and Methods" for the references to the datasets). Minimal and maximal values for each track are shown on the left in linear scale (the same scale for Figures S3-S11). Red rectangle corresponds to the position of the 6T1_L repeat cluster. Genomic coordinates are for hg38 human genome assembly.

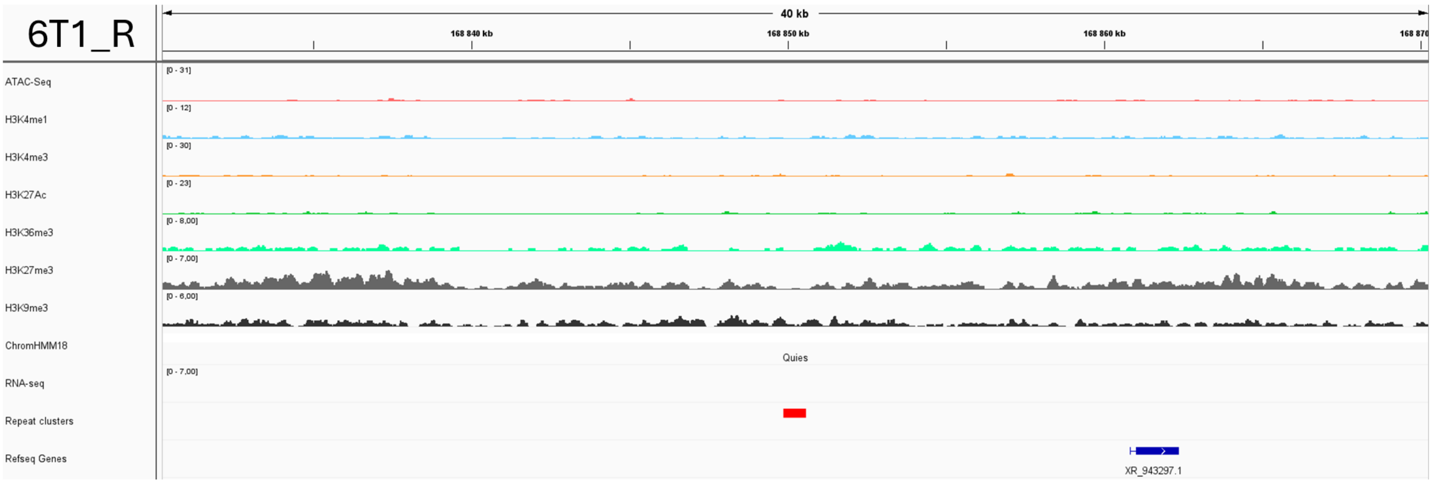

Figure S6. Epigenetic context of the 6T1_R locus (Chr6). Shown are tracks for ATAC-Seq (fold change over control), the specified histone marks (fold change over control), ChromHMM chromatin 18-state model and PolyA-plus RNA-seq (signal of unique reads). See "Material and Methods" for the references to the datasets). Minimal and maximal values for each track are shown on the left in linear scale (the same scale for Figures S3-S11). Red rectangle corresponds to the position of the 6T1_R repeat cluster. Genomic coordinates are for hg38 human genome assembly.

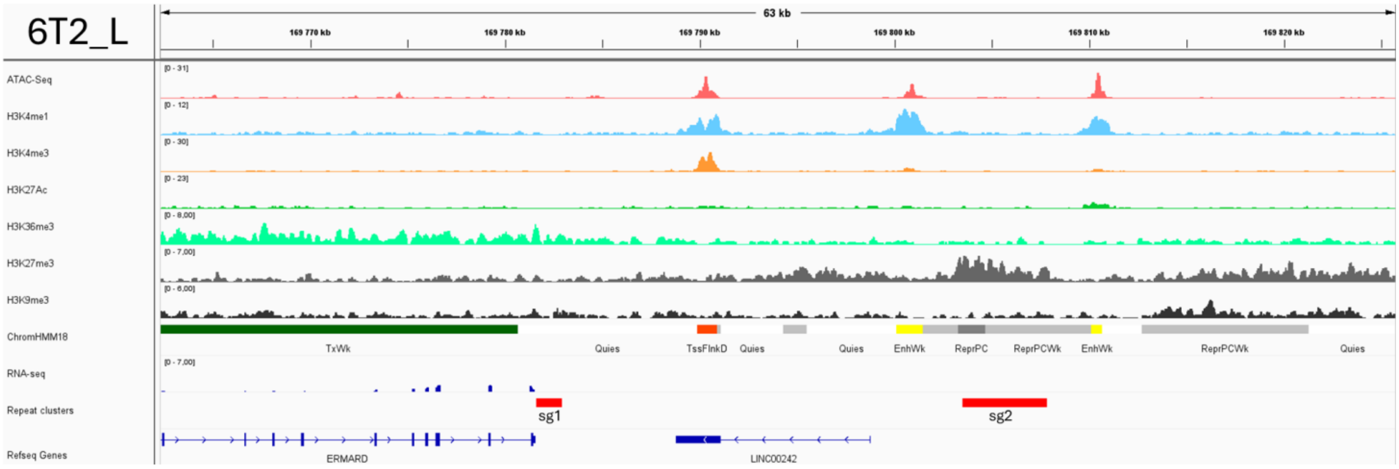

Figure S7. Epigenetic context of the 6T2_L locus (Chr6). Shown are tracks for ATAC-Seq (fold change over control), the specified histone marks (fold change over control), ChromHMM chromatin 18-state model and PolyA-plus RNA-seq (signal of unique reads). See "Material and Methods" for the references to the datasets). Minimal and maximal values for each track are shown on the left in linear scale (the same scale for Figures S3-S11). Red rectangles correspond to the positions of the 6T2_L_sg1 and 6T2_L_sg2 repeat clusters. Genomic coordinates are for hg38 human genome assembly.

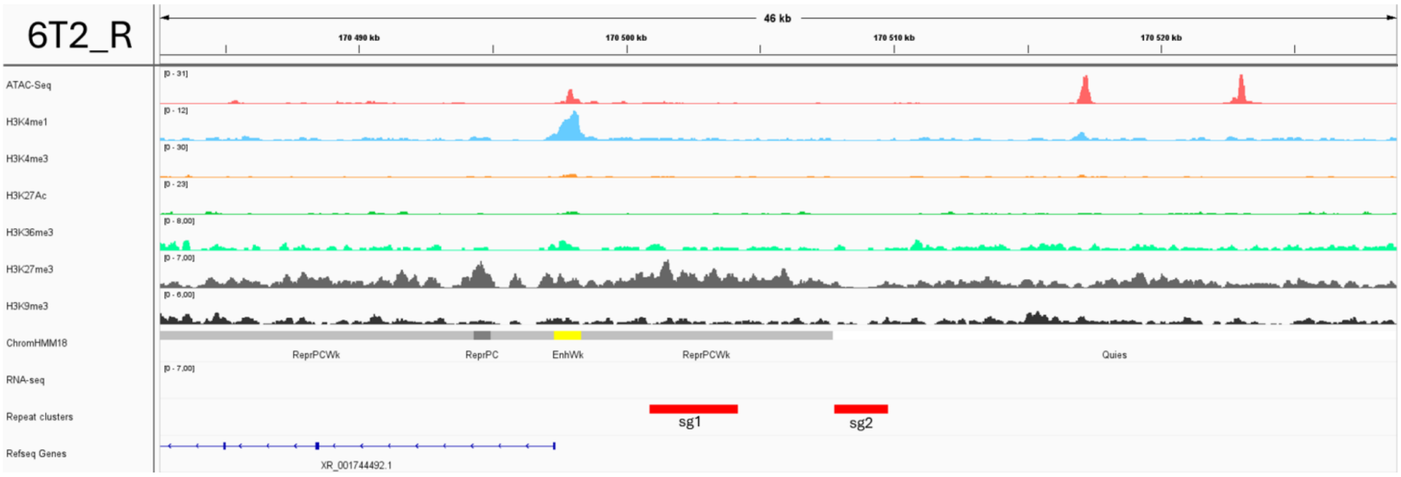

Figure S8. Epigenetic context of the 6T2_R locus (Chr6). Shown are tracks for ATAC-Seq (fold change over control), the specified histone marks (fold change over control), ChromHMM chromatin 18-state model and PolyA-plus RNA-seq (signal of unique reads). See "Material and Methods" for the references to the datasets). Minimal and maximal values for each track are shown on the left in linear scale (the same scale for Figures S3-S11). Red rectangles correspond to the positions of the 6T2_R_sg1 and 6T2_R_sg2 repeat clusters. Genomic coordinates are for hg38 human genome assembly.

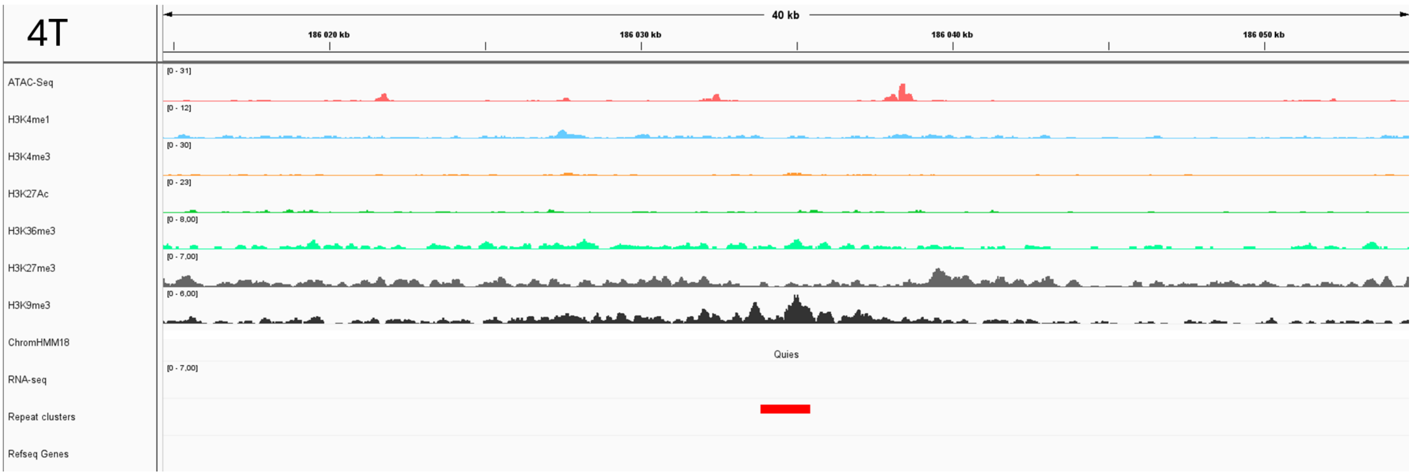

Figure S9. Epigenetic context of the 4T locus (Chr4). Shown are tracks for ATAC-Seq (fold change over control), the specified histone marks (fold change over control), ChromHMM chromatin 18-state model and PolyA-plus RNA-seq (signal of unique reads). See "Material and Methods" for the references to the datasets). Minimal and maximal values for each track are shown on the left in linear scale (the same scale for Figures S3-S11). Red rectangle corresponds to the position of the 4T repeat cluster. Genomic coordinates are for hg38 human genome assembly.

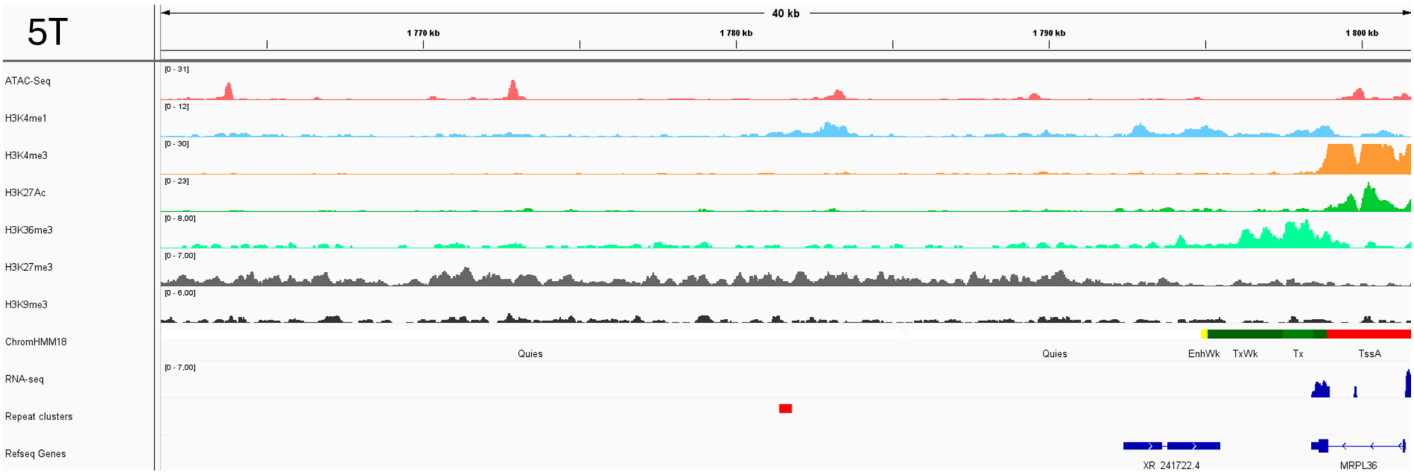

Figure S10. Epigenetic context of the 5T locus (Chr5). Shown are tracks for ATAC-Seq (fold change over control), the specified histone marks (fold change over control), ChromHMM chromatin 18-state model and PolyA-plus RNA-seq (signal of unique reads). See "Material and Methods" for the references to the datasets). Minimal and maximal values for each track are shown on the left in linear scale (the same scale for Figures S3-S11). Red rectangle corresponds to the position of the 5T repeat cluster. Genomic coordinates are for hg38 human genome assembly.

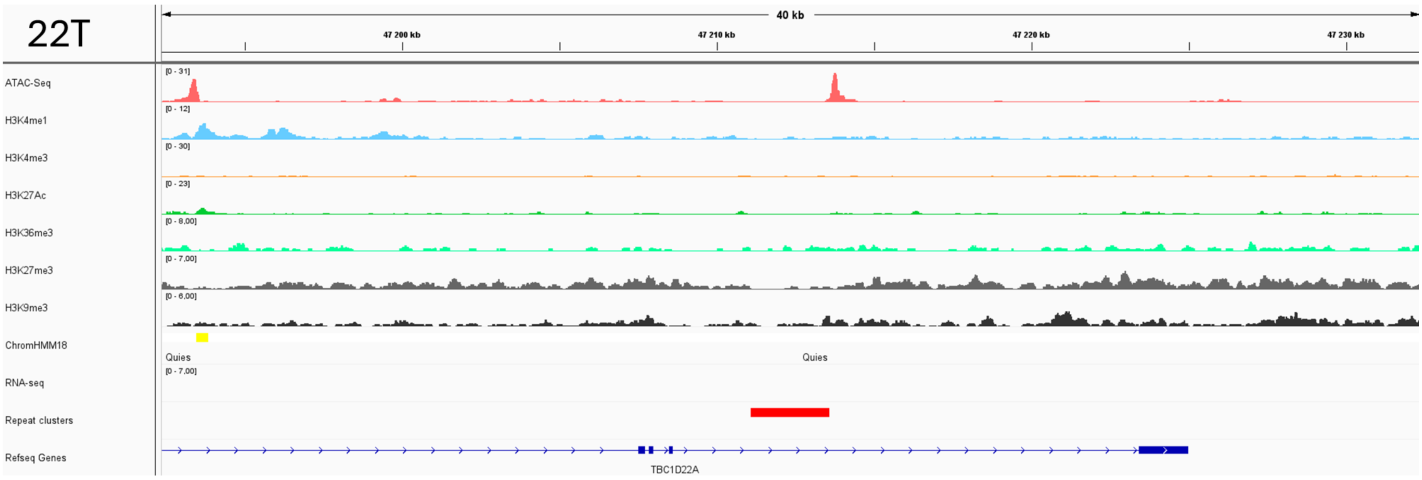

Figure S11. Epigenetic context of the 22T locus (Chr22). Shown are tracks for ATAC-Seq (fold change over control), the specified histone marks (fold change over control), ChromHMM chromatin 18-state model and PolyA-plus RNA-seq (signal of unique reads). See "Material and Methods" for the references to the datasets). Minimal and maximal values for each track are shown on the left in linear scale (the same scale for Figures S3-S11). Red rectangle corresponds to the position of the 22T repeat cluster. Genomic coordinates are for hg38 human genome assembly.

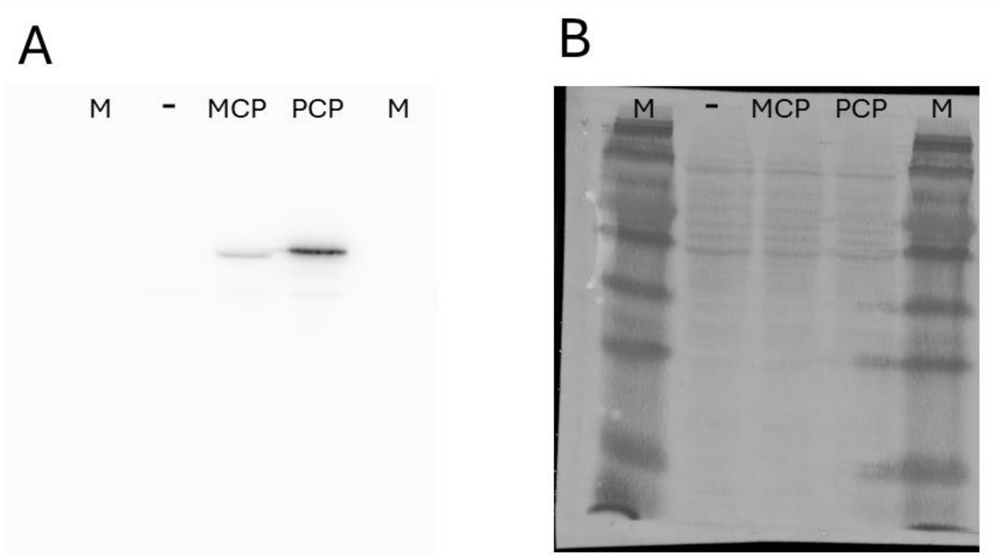

Figure S12. Raw images of the membrane (Western blot) corresponding to Figure 4A. A – ECL detection. B – Ponceau S staining. See “Material and methods” and Section 3.4 for further details.
